# Transient gene expression with CHO cells in conditioned medium: a study using TubeSpin^®^ bioreactors

**DOI:** 10.1186/1753-6561-5-S8-P38

**Published:** 2011-11-22

**Authors:** João Pereira, Yashas Rajendra, Lucia Baldi, David L Hacker, Florian M Wurm

**Affiliations:** 1 Laboratory of Cellular Biotechnology, Faculty of Life Sciences, Ecole Polytechnique Fédérale de Lausanne, CH-1015 Lausanne, Switzerland

## Background

Transient gene expression (TGE) allows rapid protein production in mammalian cells and has become an important tool in the pharmaceutical product development pipeline [1]. Polyethylenimine (PEI)-mediated, high-density transfection allowed to express recombinant proteins at yields exceeding 1 g/L in only a few weeks [2]. Although highly efficient protocols are available, volumetric scale-up of TGE is still a challenge. A major issue is the need to perform the transfection in fresh medium rather than in conditioned (spent) medium. This implies a medium exchange step just before transfection. In CHO-DG44 [3] cells we observed up to a 100-fold decrease in volumetric protein production if transfections were performed in conditioned medium, compared to fresh medium. The reasons for such a negative effect of conditioned medium on transfectability and/or protein production expression are not yet known. To study this problem we transfected CHO cells at small-scale in TubeSpin^®^ bioreactor 50 tubes using 41 different commercially available serum-free media formulations in combination with different transfection parameters and culture conditions. By comparing the transient production of a recombinant IgG antibody among the different media, we observed variation of up to 400-fold when transfecting in fresh media and up to 20-fold when using conditioned medium. The optimization of the PEI:DNA ratio allowed a significant improvement in yields of transfection in conditioned medium.

## Methods

Suspension-adapted CHO-DG44 cells [3] were routinely cultivated in TubeSpin^®^ bioreactor 50 tubes (TPP, Trasadingen, Switzerland) in ProCHO5 medium (Lonza, Vervier, Belgium) supplemented with 13.6 mg/L hypoxanthine, 3.9 mg/L thymidine, and 4 mM glutamine. The 38 media samples for transfection were provided by Excellgene SA. Conditioned medium is defined as a cell culture medium where cells have been growing for more than two days up to a density between 4-5 million cells/mL. Cell growth was accessed with the Packed Cell Volume method. The dual expression vector pXLGCHO-A3, containing the cDNAs coding for human anti-Rhesus D IgG1 heavy and light chain cloned in separate expression cassettes in a head-to-head orientation, was kindly provided by Excellgene SA [4]. Transfections are performed at a cell density of 5.5 million cells/mL at a volume of 5 mL by the direct addition of 15 µg of pDNA and 76 µg of linear 25 kDa Polyethylenimine (PEI, Polysciences, Eppenheim, Germany)

## Results

**Figure 1:**
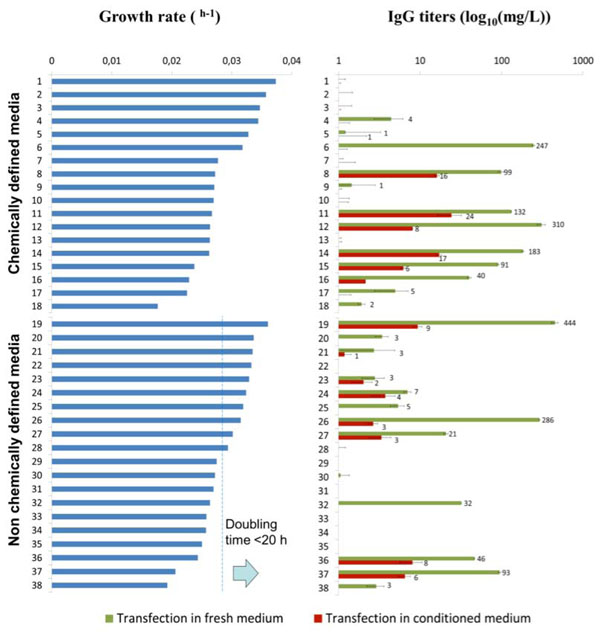
Using the TubeSpin^®^ bioreactors, 38 commercially available cell culture media were studied for their impact in cell growth and in transient protein production in a 7 day batch process at the 5 mL scale. Error < 5 % for growth rate. (n=2)

Cells were inoculated in 38 different commercially available serum free media formulations tested, and cell growth was assessed daily by the packed cell volume method [5], over 3 days of culture. We identified 16 media formulations that allowed for fast cellular growth (doubling time < 20h) up to the cell density of transfection (Figure 1). The same TGE protocol was applied in the 38 media formulations tested in a 7-day long batch process. Antibody productivity was analyzed by ELISA at day 7. Recombinant IgG production was observed only in 13 among the 38 media formulations studied, and only in 5 media titers over 100 mg/L were achieved. Transfection in conditioned media yielded in the best cases only 1/10^th^ of the production titers observed in fresh medium. Interestingly, the chemically defined media which sustained the fastest cellular growth (1-5, Figure 1) were not suitable for transient gene expression under the transfection conditions applied in this study.

In a separate study, we tested different transfection conditions that could lead to an improvement of the process yields (data not shown). We observed that increasing the PEI and DNA concentrations could improve transient gene expression titers 3-fold when transfecting in conditioned medium.

## Conclusion

This work shows that the cell culture medium has a strong impact on the transient gene expression process. We have observed that even in media formulations that sustain a very good cell growth, polycation-mediated transfection is not very efficient. When transfecting in conditioned medium with a transfection procedure optimized for fresh medium, the transient gene expression process is not satisfactory in any of the media formulation tested. We were able to improve the transfection process by changing the PEI:DNA ratio (data not shown). In order to design a robust transfection protocol that would be effective in fresh medium as well as in conditioned medium, it is necessary to understand what factors affect the transfection negatively in the latter. This study suggests that a process without a medium exchange can be designed, facilitating easier scale-up of transient gene expression.
